# Prognostic and diagnostic utility of interleukin-6 in pediatric pulmonary arterial hypertension — a case-control study

**DOI:** 10.1007/s00431-023-05413-2

**Published:** 2024-01-09

**Authors:** Mohamed Abdallah Abd El Megied, Mohammed Ahmed Abouelhassan, Eman Saad Abd El Salam Hadwa

**Affiliations:** 1 Department of Pediatrics, Faculty of Medicine, Cairo University, Cairo, Egypt; 2 Ministry of Health and Population, Cairo, Egypt

**Keywords:** Diagnostic, Interleukin-6, Prognostic, Pulmonary arterial hypertension

## Abstract

Pulmonary arterial hypertension (PAH) in pediatrics is a progressive disease with significant vascular remodeling, right sided heart failure, and death if left untreated. Elevated interleukin-6 (IL-6) level in PAH patients is taken as an independent predictor of adverse outcome including mortality. The aim of this study was to investigate and compare serum levels of IL-6 in children with PAH and healthy matched controls, and correlate between IL-6 and degree of PAH, as well as mortality. IL-6 was measured by ELISA in serum samples in 40 children with PAH (age 1–12 years) and 40 age and sex-matched healthy controls. There was a statistically significant increase in IL-6 level among PAH cases compared with the controls (1.85 ng/L vs 1.30 ng/L, *p*-value = 0.004). IL-6 at cut off point 1.45 ng/L significantly predict pulmonary hypertension in children (*AUC* = 0.685, 75% sensitivity, and 65% specificity with *p* = 0.002). There was no statistically significant association between IL-6 level and degree of PAH (*p* = 0.218). There was no statistically significant association between IL-6 level and mortality (*p* = 0.662).

*Conclusion*: IL-6 significantly predict PAH in pediatrics but there is no association between IL-6 level and degree of PAH or mortality. IL-6 may provide a less costly and less invasive method for disease detection.

**Table:** 

**What is Known:** *• Definitive diagnosis of PAH is made by right heart catheterization, while echocardiography is the gold standard for tracking the course of the disease.*	
**What is New:** *• It was assumed that children with a diagnosis of PAH would have higher serum IL-6 levels than controls. Furthermore, an adverse relationship between the blood IL-6 level and PPAH was predicted.*

**What is Known:**

*• Definitive diagnosis of PAH is made by right heart catheterization, while echocardiography is the gold standard for tracking the course of the disease.*

**What is New:**

*• It was assumed that children with a diagnosis of PAH would have higher serum IL-6 levels than controls. Furthermore, an adverse relationship between the blood IL-6 level and PPAH was predicted.*

## Background

The diagnosis of pediatric pulmonary arterial hypertension (PPAH) can be defined by a mean pulmonary arterial pressure (mPAP) of more than 20 mmHg, a pulmonary capillary wedge pressure (PCWP) of less than 15 mmHg at rest, and a pulmonary vascular resistance index (PVRI) of three wood units m^2^ or higher [1]. The prevalence of PPAH is thought to be between 20 and 64 cases per million [2].

The multifactorial causes of PPAH are highlighted by the Panama classification. These include congenital heart disease (CHD), abnormal lung growth and development, chromosomal abnormalities, sleep disorders related to breathing, chronic aspiration, and prenatal insults that lead to postnatal lung disease [3]. Compared to adult PAH, PPAH is more often caused by CHD, with fewer occurrences resulting from idiopathic pulmonary arterial hypertension (IPAH), connective tissue disease, medication exposure, or infection [4].

Definitive diagnosis of PAH is made by right heart catheterization (RHC), a procedure that carries a non-negligible risk of death, cardiac arrest, and requires mechanical circulatory support [5, 6]. While echocardiography is the gold standard for tracking the course of the disease in patients with PAH, it frequently lacks the sensitivity needed to guide treatment choices [2]. Moreover, even though measuring the 6-min walk distance (6MWD) is a useful tool for tracking the severity of an illness, it is impractical for little children [7]. Therefore, pediatrics need feasible monitoring tools [8].

It is thought that plasma cytokines could reflect the underlying lung vascular dysfunction [9], ventricular damage [10], and inflammation [11]. Pleiotropic pro-inflammatory cytokine interleukin 6 (IL-6) plays a variety of roles in immune response, inflammation, hematopoiesis, cell survival, proliferation, and apoptosis [12]. Patients with PAH were reported to have higher levels of IL-6 [13]. IL-6 is acknowledged as having a significant role in the pathobiology of PAH [14].

There are not many published studies discussing the role of IL-6 in PAH, particularly for younger age groups [4, 5]. Therefore, more research is needed to address this issue. In this study, the serum IL-6 level of children with PAH was examined and compared to that of a control group of same age and sex. We additionally examined at the association between the severity of PPAH and the serum IL-6 level. It was assumed that children with a diagnosis of PAH would have higher serum IL-6 levels than controls. Furthermore, an adverse relationship between the blood IL-6 level and PPAH was predicted.

## Methods

### Study setting

Eighty Egyptian children participated in this case-control study, which was conducted from November 2021 to April 2022 at Cairo University Children Hospital “Abu El Reesh,” Faculty of Medicine.

### Study population

The participant children were stratified into two groups; a study group “group A” and a control one “group B.” Group “A” included 40 children with PAH consecutively admitted to the pediatric intensive care units for mechanical ventilation (MV) and/or vasoactive support, either post-operative or in association with critical medical illness. Forty healthy children who matched in terms of age and sex were selected from the outpatient surgery clinic to form Group “B.”

The study’s inclusion criteria were Egyptian children with PPAH who were between the ages of 1 and 12 years. PPAH was diagnosed by echocardiographic. Patients who either passed away within 24 h of arrival or declined to participate in the trial were excluded from the study.

### Procedure

A thorough child history was obtained once informed consent was obtained. This included the patient’s age, gender, diagnosis, and PICU admission indication. Patients underwent physical examinations. This included meticulous general examination with emphasis on vital signs, anthropometric measurement (weight), chest examination (chest deformity, respiratory rate, auscultation for breath sounds, and any additional sounds), abdominal examination (hepatomegaly and ascites in severe cases), and extremities for lower limb edema in severe cases.

Detailed systemic examination was conducted to verify the inclusion and exclusion criteria and level of respiratory and cardio vascular support. Detailed cardiovascular examination was performed including pulse, blood pressure, inspection of pericardium (pericardial bulge, visible pericardial pulsation, scars, and dilated veins), palpation for (apex beat, parasternal heave, and thrills), and auscultation for (heart sounds and any additional murmurs).

Every enrolled patient was subjected to chest X-ray (CXR), and echocardiographic assessment of mean pulmonary artery pressure (mPAP), right ventricular diameter (apical 4 chamber view) and right ventricular outflow tract fraction of shortening (RVOTFS) (M — mode from the parasternal short axis view at aortic valve level). The right ventricular diastolic function was assessed using pulsed trans tricuspid Doppler, which yielded the tricuspid E/A ratio. The peak early filling velocity is represented by an E wave, and the peak late filling velocity by an A wave. Tricuspid annular plane systolic excursion (TAPSE) as a scoring system was used with non-invasive Doppler echocardiography to determine right ventricular (RV) function. PAH classification includes three categories, mild PAH (pulmonary arterial systolic pressure (PASP) = 36–45 mmHg), moderate PAH (PASP = 45–60 mmHg), and severe PAH (PASP > 60 mmHg).

The following laboratory tests were conducted. Each participant had 5 mL of venous blood collected from them using a sterile syringe. The withdrawn blood sample was distributed between two sterile vacutainers: one EDTA vacutainer for complete blood picture (CBC) testing using automated blood counter (Sysmex XN-1000) and another serum vacutainer for separating serum after sample centrifugation. The separated serum was used for C-reactive protein (CRP) testing in addition to measuring serum IL-6 by commercial enzyme-linked immunosorbent assay (ELISA) technique (Human IL-6 ELISA Kit, Catalog No: SG-10267, made in China) with reference range (0.2–8 ng/L).

### Study outcomes

Children with PAH and healthy matched controls were compared for their levels of IL-6. Along with the degree of PAH, the study group’s outcome and serum IL-6 level were also correlated.

### Data management and statistical analysis

Data including participant history, clinical examination, laboratory investigations, and outcome measures were gathered, coded, and analyzed. Analysis was conducted using Statistical Package for the Social Sciences (SPSS version 20.0). For parametric numerical data, the measures are mean, standard deviation (± SD), and range; for non-parametric numerical data, the metrics are median and inter-quartile range (*IQR*). The statistical significance of the differences in non-parametric variables between the two groups was evaluated using Mann–Whitney. ROC curve was constructed with area under curve analysis performed to detect best cutoff value of IL-6 for detection of cases. *P*-values of less than 0.05 were considered as statistically significant.

## Results

After screening 180 admittents to the PICU at Cairo University’s Children Hospital from November 2021 to April 2022, 50 patients were found to have PAH. Just forty patients who volunteered to participate in the study were included in the analysis, along with forty healthy controls who were chosen from the surgical and follow-up clinics with permission from their caretakers. Their age and gender matched that of the patient group.

Group ‘A’ had mean age of 5.2 years (range, 1–12 years), with 19 (47.5%) males and 21 (52.5%) females. There were 19 (47.5%) males and 21 (52.5%) females in group ‘B,’ with mean age of 5.26 years (range, 1–12 years). Age and sex did not significantly differ between both groups (*p* = 0.9 and 0.99, respectively). According to the anthropometric evaluation of the enrolled patients, group A mean weight was 16.22 kg, and group B was 22.16 kg. The weight was significantly different between both groups being lower in group A (*p* = 0.009).

Disease-associated pulmonary artery hypertension (DAPAH) with congenital heart diseases was the most common diagnosis in group A (40%). DAPAH with cystic fibrosis (17.5%), DAPAH with pneumonia (12.5%), DAPAH with hematological diseases (10%), idiopathic pulmonary artery hypertension (IPAH) (7.5%), DAPAH with septic shock (7.5%), DAPAH with restrictive cardiomyopathy (2.5%), and DAPAH with heart failure (2.5%) were the other diagnoses.

Regarding the laboratory investigations in group A, it was found that the mean values of HB, TLC, PLT, and CRP were 11.50 g/dL, 10.30 cells/µL, 275.00 cells/µL, and 10.40 mg/L, respectively. In comparison to group B, group A had a statistically significant higher IL-6 level (1.85 ng/L vs. 1.30 ng/L, *p* = 0.004). On echocardiographic evaluation, the mean values of mPAP, RVOT FS%, FS%, EF%, and TAPSE were 50.95 ± 14.53 mmHg, 36.03 ± 10.64%, 39.03 ± 8.12%, 67.85 ± 11.63%, and 16.83 ± 2.09 mm, respectively (Table 1). On stratification of the degree of PAH by echocardiography, 17 (42.5%) patients had mild PAH, 14 (35%) severe, and 9 (22.5%) moderate PAH.

**Table 1:** IL-6 level and echocardiography findings in the study

	**Cases**				**Controls**				***p* value**
	***Mean***	***SD***	**Min.**	**Max.**	***Mean***	***SD***	**Min.**	**Max.**	
**Level of IL-6**	3.47	4.09	0.90	14.70	1.97	1.89	1.00	11.50	0.004
**RVOT FS%**	36.03	10.64	15.00	45.00	43.18	1.77	39.00	45.00	0.001
**FS%**	39.03	8.12	10.00	55.00	69.88	7.07	55.00	79.00	< 0.001
**EF%**	67.85	11.63	22.00	90.00	40.55	3.84	33.00	45.00	< 0.001
**TAPSE (mm)**	16.83	2.09	12.00	20.00	15.60	4.42	10.00	26.00	0.098
**mPAP (mmHg)**	50.95	14.53	33.00	95.00	10.00	2.65	5.00	15.00	< 0.001

*RVOT* right ventricle outflow tract, *FS* fraction segment, *EF* ejection fraction, *TAPSE* tricuspid annular plane systolic excursion, *mm* millimeters, *mPAP* mean pulmonary artery pressure, *mmHg* millimeters of mercury

In the case group, there was no statistically significant correlation between CRP level and either of IL-6 level (*r* =  − 0.058, *p* = 0.722) or degree of PAH (*r* = 0.291, *p* = 0.069) (Table 2). Table 3 shows that there was no significant correlation (*p* = 0.218) between IL-6 and the severity of PAH. Furthermore, there was no discernible relationship between the IL-6 level and the other laboratory, clinic, and echocardiographic results (Table 4).

**Table 2:** The correlation between CRP, IL-6 level, and degree of PAH

		**Level of IL-6 (ng/L)**	**Degree of PAH**
**CRP (mg/L)**	**Correlation coefficient**	− 0.058	0.291
	***p* value**	0.722	0.069
	***N***	40	40

*IL-6* interleukin-6, *PAH* pulmonary arterial hypertension, *CRP* C reactive protein, *mg/L* milligrams per liter, *ng/L* nanogram per liter

**Table 3:** Correlation between IL-6 levels and severity of PAH

		**Level of IL-6 (ng/L)**				***p* value**
		***Mean***	***SD***	**Min.**	**Max.**	
**Degree of PAH**	**Mild** (*PASP* = 36–45 mmHg)	5.32	5.64	1.10	14.70	0.218
	**Moderate** (*PASP* = 45–60 mmHg)	2.26	0.76	1.60	3.80	
	**Severe** (*PASP* > 60 mmHg)	2.01	1.66	0.90	7.60	

*PAH* pulmonary artery hypertension, *IL-6* interleukin-6/, *ng/L* nanogram per liter

**Table 4:** Correlation between IL-6 and other parameters in children diagnosed with PAH

	**Correlation coefficient**	***p* value**
**Age (years**)	0.051	0.753
**Weight (kg**)	0.003	0.983
**HR (beat/min)**	0.117	0.473
**RR (cycle/min)**	− 0.119	0.466
**SBP (mmHg)**	0.020	0.900
**DBP (mmHg)**	0.170	0.295
**HB (g/dL)**	0.215	0.188
**TLC (cells/µL)**	− 0.048	0.773
**PLT (cells/µL)**	0.203	0.216
**RVOT FS%**	0.180	0.267
**FS%**	0.062	0.706
**EF%**	0.103	0.526
**TAPSE (mm)**	0.073	0.653

*HR* heart rate, *RR* respiratory rate, *SBP* systolic blood pressure, *DBP* diastolic blood pressure, *HB* hemoglobin, *TLC* total leukocyte count, *PLT* platelets, *CRP* C-reactive protein, *mPAP* mean pulmonary artery pressure, *RVOT* right ventricle outflow tract, *FS* traction segment, *EF* ejection fraction, *TAPSE* tricuspid annular plane systolic excursion, *ng/L* nanogram per liter, *mmHg* millimeter mercury, *mm* millimeter, *beat/min* heart beat per minute, *cells/µL* cells per microliter, *kg* killogram

**Fig. 1:**
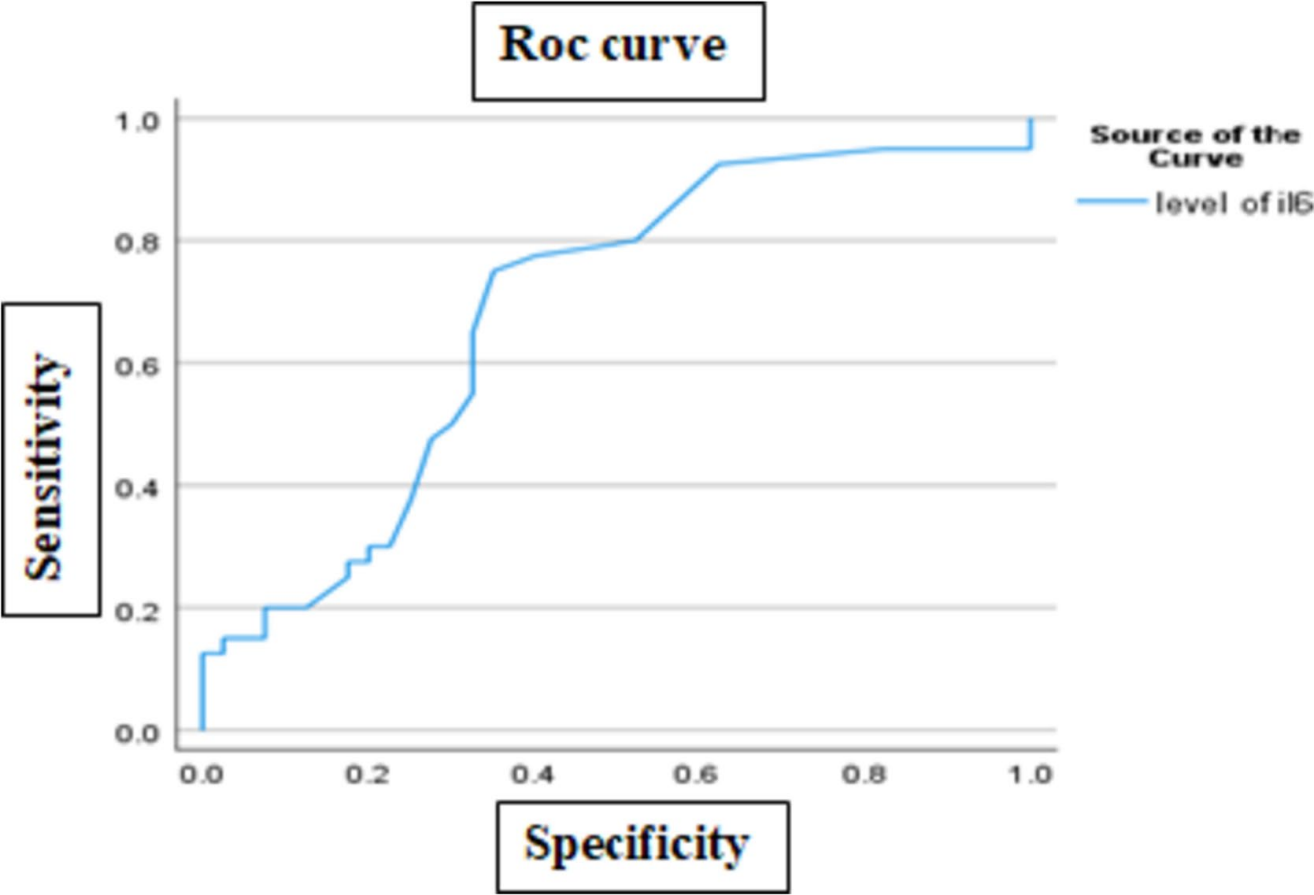
ROC curve analysis of IL-6 for diagnosis of PAH

ROC curve analysis showed that IL-6 at cut off point 1.45 ng/L significantly predict PAH in patient (*p* = 0.002, *AUC* = 0.685, 75% sensitivity, and 65% specificity (Fig. 1).

Of the 40 patients enrolled in this study; 26 (65%) were readmitted; 9 (22.5%) were discharged; 5 (12.5%) died with a total of 35 (87.5%) who survived; and 5 (12.5%) died. According to the prognostic value of IL-6 in PAH, the study showed that there was no statistically significant association between IL-6 level and mortality (*p* = 0.662) (Table 5).

**Table 5:** Relation of IL-6 level and the outcome

		**Level of IL6**				***p* value**
		***Mean***	**Standard deviation**	**Minimum**	**Maximum**	
**Outcome**	Survivors	3.67	4.33	0.90	14.70	0.662
	Non-survivors	2.08	0.56	1.50	3.00	

## Discussion

PAH is a progressive disease in both pediatrics and adults that results in significant vascular remodeling due to increased resistance in the pulmonary circulation. It causes right-sided heart failure and death if left untreated [6]. In our study, the 40 children with PAH could be representative for the estimated 20–64 cases per million, and consecutively the obtained data could be a corner stone for further studies aiming at early, effective, and proper management of PPAH.

Right heart catheterization (RHC) is considered the gold standard for diagnosing pulmonary arterial hypertension (PAH). During this procedure, the Swan Ganz catheter is used to monitor the pulmonary artery pressure directly and to determine the pulmonary vascular resistance index (PVRI) [15]. In addition to being intrusive, RHC is also expensive, time-consuming, and skill-dependent. Regretfully, it is limited to a few locations throughout Africa. In middle- and low-income nations, the transthoracic echocardiography has been suggested as the mainstay for diagnosing PAH [16].

The most often used methods for evaluating disease progression are hemodynamic tests and the 6-min walk distance, yet neither of these has been fully established in the pediatric population and has practical limitations. Due to the limits in outcome and prognostic variables, there is a need for alternatives that are easy to evaluate in children and that are dependable, non-invasive, objective, and affordable [17].

PAH is characterized by endothelial dysfunction and subsequent remodeling of the pulmonary vasculature, which has been linked to the pro-inflammatory cytokine IL-6 [14]. More research has been done on IL-6 in adults as a potential therapeutic target and an early pathobiological biomarker of disease development [18]. Although IL-6 has not been extensively researched in children, a study of pediatric PAH incorporating a broad cytokine panel and univariate analysis found that IL-6 was linked to unfavorable outcomes, indicating that IL-6 may have some use as a prognostic marker in pediatric patients [13].

In the current study, with regard to age and gender, there were no appreciable differences between the two groups. Hence, their ages and sexes were matched. Meanwhile, there was statistically significant difference between both groups regarding weight being lower in group A. This could be explained by the fact that the chronic inflammatory status among patients of group A resulted from chronic disease. Additionally, recurring hospital admission affects the nutritional status and consequently the weight. In 75% of the patients, tachypnea was evident. This result is in line with the finding of Ezekian et al. [19]. This might be due to lung pathology that occurs due to lung oligemia and lung parenchymal affection due to PAH.

The present study revealed a statistically significant rise in IL-6 levels between group A and group B. This is consistent with Oz-Tuncer [20]. They found that the levels of IL-6 were significantly increased in children with PAH. Additionally, our results are consistent with those published by Humbert [22] and Selimovic [21]. They conducted their studies on adult patients with PAH. According to their findings, PAH patients’ IL-6 levels were noticeably greater than those of controls. The observed rise in interleukin-6 (IL-6) levels in both pediatric and adult age groups may suggest a pathogenic function of IL-6 in the course of disease [23]. Our results do not match the information provided by Hoeper et al. [24]. The latter study did not discover a statistically significant rise in serum IL-6 levels in PAH patients. This difference could be attributed to different sample size, age of the studied groups, and stages of the disease.

The receiver operating characteristic (ROC) curve for IL-6 was plotted in order to assess the diagnostic accuracy of IL-6 in PPAH. It revealed that the sensitivity and specificity of IL-6 at cutoff point 1.45 ng/L were 75% and 65%, respectively, in discriminating PPAH from non-PPAH. This indicates that IL-6 has a diagnostic performance for PPAH. Our finding is supported by what was reported by Oz-Tuncer [20]. They found that IL-6 had better diagnostic performance.

There was no relationship found in the current research between the level of PAH and IL-6. This is in agreement with Prins’ findings [25]. They reported that the level of serum IL6 and mPAP did not significantly correlate. There are several possible explanations for these observations. First, IL-6 is more crucial for the start of pulmonary vascular remodeling than for the ongoing phenotypic progression of the disease [25]. Secondly, it is possible that IL-6 contributes to the pathophysiology of PAH rather than being a reflection of right ventricular function [26]. Moreover, hypoxia is necessary to encourage a more severe form of pulmonary hypertension, whereas IL-6 overexpression only causes moderate hypertension [27]. We should take in consideration that IL-6, promptly and transiently produced in response to infections and tissue injuries, contributes to host defense through stimulation of acute phase responses, hematopoiesis, and immune reactions [14]. So, clinical assessment is mandatory and laboratory finding will never replace clinical evaluation.

The reported findings are against those of Chen [18]. The latter reported that each log-unit higher IL-6 is significantly associated with higher mPAP over time [18]. The difference between their findings and ours could be attributed to the difference in time between hemodynamic measures and serum samples. Furthermore, the majority of patients had been treated for PAH at the time of enrollment, which may have had an impact on the disease’s actual serum levels of IL-6 [18].

In the current study, there was no association between IL-6 level and mortality. This is consistent with the study conducted by Cracowski [28] and Koudstaal [23]. According to their findings, there was no relationship between IL-6 and survival in any of the PAH subgroups. Additionally, according to Koudstaal et al. [23] IL-6 did not significantly differ between PAH patients with a 3-year survival rate of less than three and those with a higher rate. The lack of a correlation between IL-6 and death may be explained by variations in pulmonary vascular disease, which did not account for the decreased RV function in patients with elevated IL-6 blood levels [25].

This is against what was reported by Duncan [13]. They found that IL-6 level was significantly and univariately associated with the occurrence of adverse events [13]. Furthermore, C-reactive protein (CRP) and IL-6 were both discovered by Heresi et al. to be univariate predictors of mortality [29]. A ninefold increase in mortality is predicted by an IL-6 cut-off value of ≥ 4.7 pg/mL, which has 86% sensitivity and 72% specificity.

Our hypothesis is validated by the results, which show that IL-6 has a high sensitivity and specificity for PPAH discrimination. IL-6 may help in early diagnosis and treatment initiation; however, clinical assessment is mandatory as laboratory evaluation will never replace clinical evaluation. It is recommended to measure circulating IL-6 for earlier management of PPAH. It is also recommended to conduct more studies on large sample sizes to understand IL-6 effect on PAH in pediatric population and focus on criteria for time for IL6 sampling to identify the effect of IL6 level in disease progression.

One of the study’s strengths is that it is one of the few on IL-6 in pediatrics, and it discovered a noteworthy rise in IL-6 in kids with PAH. However, it has several limitations. These include small sample size, and the fact that echocardiography was used to diagnose PAH (though RHC is available in few centers). The enrollment of incident and prevalent patients may have had an impact on the total IL-6 levels.

## Conclusion

In comparison to controls, IL-6 levels are higher in PPAH patients. IL-6 may significantly predict PAH in children at a cutoff point 1.45 ng/L with 75% sensitivity, and 65% specificity. There are no associations between IL-6 level and degree of PAH and mortality. For the identification of disease, IL-6 might offer a less expensive and invasive approach.

## Data Availability

All the data are available upon request.

## Abbreviations

6MWD: 6-Min walk distance
*AUC*: Area under curve
beat/min: Heartbeat per minute
CBC: Complete blood picture
cells/µL: Cells per microliter
CHD: Congenital heart disease
CRP: C-reactive protein
CXR: Chest X-ray
DAPAH: Disease-associated pulmonary artery hypertension
DBP: Diastolic blood pressure
EDTA: Ethylene-diamine-tetra-acetic
EF: Ejection fraction
ELISA: Enzyme-linked immunosorbent assay
FS: Fraction segment
g/dL: Gram per deciliter
HB: Hemoglobin
HR: Heart rate
IL-6: Interleukin-6
IPAH: Idiopathic pulmonary arterial hypertension
*IQR*: Inter-quartile range
kg: Kilogram
mg/L: Milligrams per liter
mm: Millimeters
mmHg: Millimeters of mercury
mPaP: Mean pulmonary arterial pressure
MV: Mechanical ventilation
ng/L: Nanogram per liter
PAH: Pulmonary arterial hypertension
PAH: Pulmonary artery hypertension
PCWP: Pulmonary capillary wedge pressure
PICU: Pediatric intensive care unit
PLT: Platelets
PVRI: Pulmonary vascular resistance index
RHC: Right heart catheterization; right ventricle outflow tract
ROC: Receiver operating characteristic
RR: Respiratory rate
RVOT: RVOTFS: right ventricular outflow tract fraction of shortening
SBP: Systolic blood pressure
*SD*: Standard deviation
SPSS: Statistical Package for the Social Sciences
TAPSE: Tricuspid annular plane systolic excursion
TLC: Total leukocyte count
